# Bismuth Infusion of ABS Enables Additive Manufacturing of Complex Radiological Phantoms and Shielding Equipment

**DOI:** 10.3390/s17030459

**Published:** 2017-02-24

**Authors:** Justin Ceh, Tom Youd, Zach Mastrovich, Cody Peterson, Sarah Khan, Todd A. Sasser, Ian M. Sander, Justin Doney, Clark Turner, W. Matthew Leevy

**Affiliations:** 1 Department of Biological Sciences, University of Notre Dame, 100 Galvin Life Science Center, Notre Dame, IN 46556, USA; jceh@nd.edu (J.C.); Zachary.Mastrovich.2@nd.edu (Z.M.); skhan6@nd.edu (S.K.); isander@alumni.nd.edu (I.M.S.); jdoney@nd.edu (J.D.); 2 Turner MedTech Inc., 1119 South 1680 West, Orem, UT 84058, USA; tyoud@turnerinnovation.com (T.Y.); cpeterson@turnerinnovation.com (C.P.); cturner@turnerinnovation.com (C.T.); 3 Notre Dame Integrated Imaging Facility, University of Notre Dame, Notre Dame, IN 46556, USA; tsasser@nd.edu; 4 Department of Chemistry and Biochemistry, University of Notre Dame, 236 Nieuwland Science Hall, Notre Dame, IN 46556, USA; 5 Harper Cancer Research Institute, University of Notre Dame, 1234 N Notre Dame Avenue, South Bend, IN 46617, USA

**Keywords:** 3D printing, imaging phantoms, CT scans, medical imaging, radiopacity, multi-material, additive manufacturing

## Abstract

Radiopacity is a critical property of materials that are used for a range of radiological applications, including the development of phantom devices that emulate the radiodensity of native tissues and the production of protective equipment for personnel handling radioactive materials. Three-dimensional (3D) printing is a fabrication platform that is well suited to creating complex anatomical replicas or custom labware to accomplish these radiological purposes. We created and tested multiple ABS (Acrylonitrile butadiene styrene) filaments infused with varied concentrations of bismuth (1.2–2.7 g/cm^3^), a radiopaque metal that is compatible with plastic infusion, to address the poor gamma radiation attenuation of many mainstream 3D printing materials. X-ray computed tomography (CT) experiments of these filaments indicated that a density of 1.2 g/cm^3^ of bismuth-infused ABS emulates bone radiopacity during X-ray CT imaging on preclinical and clinical scanners. ABS-bismuth filaments along with ABS were 3D printed to create an embedded human nasocranial anatomical phantom that mimicked radiological properties of native bone and soft tissue. Increasing the bismuth content in the filaments to 2.7 g/cm^3^ created a stable material that could attenuate 50% of ^99m^Technetium gamma emission when printed with a 2.0 mm wall thickness. A shielded test tube rack was printed to attenuate source radiation as a protective measure for lab personnel. We demonstrated the utility of novel filaments to serve multiple radiological purposes, including the creation of anthropomorphic phantoms and safety labware, by tuning the level of radiation attenuation through material customization.

## 1. Introduction

The field of medical imaging spans the preclinical and clinical research space with several imaging modalities including magnetic resonance imaging (MRI), ultrasound (US), and three-dimensional X-ray computed tomography (CT) [1]. CT scanners, which began clinical use in the 1970s, have seen tremendous technical advancement over the last four decades, with decreasing scan times and increasing fields of view and image quality [2]. As innovation continues, physical models known as phantoms, which can directly mimic human tissues, are employed during the design, optimization, validation, and comparison processes for medical imaging systems. These phantoms can be used in place of actual human tissue to precisely test, calibrate, and classify parameters of the imaging systems, such as spatial resolution and imaging noise level [3]. Imaging phantoms are also an important teaching tool to educate and train new users of medical imaging modalities [4]. In order to fabricate phantoms ideally suited for validation and training purposes, it is important to use materials that match physical parameters, such as radiopacity, of the tissue they will be representing. The first portion of the work presented here focuses on creating a phantom material that matches the radiopacity of bone and can be processed via additive manufacturing.

Additive manufacturing, also known as three-dimensional (3D) printing, is a process of building objects in an incremental, layer-by-layer fashion. It has recently emerged as a powerful tool in the creation of complex parts and models, especially those with internal cavities and intricacies. Three-dimensional printers are becoming increasingly ubiquitous due to new applications and their relatively low price [5]. These machines can now synthesize objects using a range of input materials including polymers [6], metals [7], and ceramics [8], while recent innovations have even used this technology for tissue engineering applications [9,10]. Some types of 3D printers use multiple heads or output ports to enable composite structures to be created from multiple materials with varying properties, such as hardness, color, or radiodensity.

The recent developments of consumer-grade, multi-material additive machines have expanded the potential use of 3D-printed, anatomically accurate phantoms as teaching tools. The ability to produce customized and cost-effective physical models of anatomical parts is a promising resource for the training of medical personnel. The use of physical models has been shown to be advantageous when learning anatomical spatial relationships [11]. The ability to print multi-material phantoms, coupled with the conversion of CT or MR images to 3D-printable files, allows for complete customization of phantoms. Body part replicas with tailored deformities can be printed with soft and hard tissue components for analysis under various imaging modalities. For example, CT data of a patient with a brain tumor has been utilized to 3D print an anthropomorphic model to train neurosurgeons in performing craniotomies [12]. Salmi used additive manufacturing to create an array of preoperative anatomical models, including a heart model and orthopedic models of the knee, backbone, and pelvis [13]. Tuomi et al. classified 3D-printed medical devices into five different classes, including educational models, and published an online repository for information and case studies associated with medical additive manufacturing [14]. Importantly, errors in the anatomical models can occur if there are process issues between collecting the imaging data and converting it to a printable template, thereby necessitating careful communication, quality control standards, and measurements of the resulting model to ensure anatomical accuracy [15]. Developing such models provides a relatively cheap and convenient alternative to relying on cadavers for education and training.

While previous work has been done in the space of custom 3D printing phantoms for medical imaging calibration and training [16,17,18,19,20,21], or even developing new 3D printing methods for fabricating mimetic optical phantoms [22], this work focuses on developing new filaments to emulate the radiopacity of the native tissues of interest during X-ray CT imaging. Leary et al. provide a thorough review of the requirements and challenges of 3D printing radiation dosimetry phantoms, including a discussion of the radiation interaction properties of commercially available 3D printing materials [23]. Since many common additive manufacturing filaments, particularly polymers, provide little radiation attenuation due to their low radiodensity, custom bismuth-infused acrylonitrile butadiene styrene (ABS) filaments were generated, allowing for a tunable radiopacity level that scaled with the concentration of the incorporated metal. This property is critical when creating an imaging phantom to ensure a CT scan yields an accurate representation of the target anatomy. The patent-pending ABS-bismuth composite filaments, commercialized under the name “GMASS,” were designed for 3D printing high-density components with adjustable radiopaque properties. By tuning the bismuth concentration, filaments with a radiopacity similar to bone were fabricated and used to 3D print a multi-material nasocranial phantom derived from patient CT scan data. An increased bismuth concentration was employed to 3D print a sample tube rack with improved radiation attenuation properties, demonstrating the customization of protective personnel safety equipment. These filaments had radiopacity properties that were not were not available from an array of commercial 3D printing filaments measured.

## 2. Materials and Methods

### 2.1. Commercial 3D Printing Filaments

Filaments for the material survey were commercially sourced as follows: Matter Hackers (Foothill Ranch, CA, USA) supplied: Bendlay Product Number 12001, IGUS Product Number 45017002, Laybrick Product Number 858029, Bismuth Product Number 42077004, Glow ABS Product Number 17957008, and Glow PLA Product Number 12002. Woodfill Product Number 8719033555037, Copperfill Product Number 8719033555211, and bronzefill Product Number 8719033555211 were ordered from Colorfabb (Belfeld, The Netherlands).

### 2.2. Preparation of ABS-Bismuth Composites of Varied Concentration

The filaments for this study were manufactured by Turner MedTech (Orem, UT, USA). The standard ABS-bismuth GMASS product has a density of 2.7 g/cm^3^. However, for the purposes of this study several filaments were manufactured that varied in density over the range of 2.5 g/cm^3^ to 1.2 g/cm^3^. To make the various concentrations, GMASS filaments were reground into pellets that were equally divided by weight into containers in which base ABS resin was added to achieve the desired filament density. Each container of pellets was well mixed and then sequentially processed through a screw extruder starting from the least desired density to the greatest. The extruded filament was then divided into sections and density samples were taken to characterize the filament and ensure that each met the requirements of this study.

### 2.3. Preclinical CT Acquisition and Reconstruction of Filaments

CT scan images of the individual filaments were acquired using an Albira CT system (Bruker, Billerica, MA, USA). The X-ray source settings were a 400 μA current at a 45 kV voltage with a 0.5 mm aluminum filter in the X-ray path for hardening. Circular scans of the filaments were taken with 400 projections per scan and a field of view (FOV) of 64 mm, and reconstructed with a 250 µm isotropic voxel size using the Albira Suite 5.0 Reconstructor, with “Filtered Back Projection” (FBP) algorithm calibrated to output data in Hounsfield Units (HU). Following the scan, data was opened in its native microPET file format and opened with PMOD software for further analysis.

### 2.4. Clinical CT Acquisition of Filaments

Images of the filaments were acquired using a clinical NeuroLogica CereTom CT scanner (NeuroLogica, Danvers, MA, USA). Data was collected using an axial test protocol with 100 kV source and current of 3 mA, with imaging resolution timing of 6 s. For each scan, the coverage length was 230 mm, with 184 images taken over a 138 s scan time to yield a reconstructed data set with voxel size of 0.5 mm × 0.5 mm × 0.625 mm and calibrated to HU. Following the scan, data was exported as a DICOM format file and imported to PMOD software for further analysis.

### 2.5. Radiodensity Measurement of CT Image Data

Pre-clinical or clinical CT data was opened in PMOD software (V 3.2, PMOD Technologies LLC, Zurich, Switzerland) for Volume of Interest (VOI) measurement of average HU (with standard deviation of voxel density) from each filament. Data were recorded and plotted in Graphpad Prism (Graphpad Software Inc., La Jolla, CA, USA).

### 2.6. 3D Printing

#### 2.6.1. Multimaterial Phantom

A de-identified, clincial CT imaging data set of a patient skull and sinuses (with signed patient auhorization for release) was acquired and reconstructed with a 0.4 mm isotropic voxel via a MiniCAT (Xoran Technologies, Ann Arbor, MI, USA) CT and associated software. Image files were imported into PMOD 3.2, and the volume to be printed containing the head was cropped by creating a volume of interest and masking the image outside the defined volume. The resulting image file was saved in nifti format and imported into 3D Slicer [24]. A threshold of 180 was applied to isolate bone tissue and a second threshold of −200 was applied to generate surface maps using the greyscale modelmaker tool. The surface maps were then exported as STL files. These files were opened in NetFabb (Autodesk, San Rafael, CA, USA) and extraneous portions of the images and degenerate faces were removed. Fine portion removal was done in MeshLab (ISTI–CNR). The surface maps were then exported as STL files to be uploaded for printing. The STL files were then imported independently into MakerBot Desktop’s (MakerBot Industries, New York, NY, USA) simulated build platform. Once in MakerBot Desktop the models were carefully aligned with one another to accurately reflect the full CT data. The 3D printer used for producing this particular phantom was the MakerBot Replicator 2X (MakerBot Industries) with a 0.4 mm nozzle diameter, 11 μm XY plane precision, 2.5 μm Z position precision, and a printing layer height of 0.2 mm. The soft tissue model was printed with native ABS using two perimeter shells and 15% hexagonal infill, while the bone model was printed using 1.2 g/cm^3^ ABS-bismuth composite with four perimeter shells and 15% infill. The models were printed simultaneously on a heated build plate using duel extruders without support structures to provide an accurate mimic of the CT scan.

#### 2.6.2. Squares and Example Personnel Protective Equipment

A 16-position test tube rack was printed solid using the 2.7 g/cm^3^ ABS-bismuth composite on the MakerBot Replicator 2X as noted above. Attenuation squares of 5 cm × 5 cm were created of varied thickness (1, 2, and 3 mm) using Autodesk Inventor, and printed on the MakerBot Replicator 2X in both native ABS and 2.7 g/cm^3^ ABS-bismuth composite.

### 2.7. 3D-Printed Model Accuracy Measurements

CT scans of the 3D-printed multimaterial head phantom were acquired using a clinical NeuroLogica CereTom CT scanner using the same acquisition parameters as used for measuring the ABS-bismuth filaments. The radiodensity was measured at five distinct regions of the phantom, all in areas corresponding to bone tissue. The radiodensity of the mimetic bone in these regions was compared to the measured radiodensity at the same locations in the original patient CT scan.

The head phantom was measured using a digital caliper at eight points, encompassing both the soft and bone tissue portions. These measurements were compared with distance measurements taken on the digital DICOM file of the patient CT scan data. The absolute percent difference in measurements was recorded at each of the eight measurement points.

### 2.8. Radiation Attenuation Measurements

Radiation attenuation experiments for comparison of 2.7 g/cm^3^ ABS-bismuth relative to native ABS were conducted using ^99m^Technetium (^99m^Tc) pertechnetate sourced from a local Cardinal Health (South Bend, IN, USA) nuclear pharmacy. Sources were diluted to 390, 180, 87, and 41 µCi activities in 100 µL of solution, each of which was measured immediately prior to experimentation using a dose calibrator (AtomLab 400, Biodex Medical Systems Inc., Shirley, NY, USA). Gamma emission from each source was captured using an Xtreme 4MP image station (Bruker Preclinical Imaging, Billerica, MA, USA) fitted with a radioisotopic phosphor screen and sensitive CCD chip cooled to −65 °C. Images were acquired using a 100 mm FOV, binning 4 × 4, and 10 s exposure time. Each point source was imaged thirteen times: without any block, and then at 1, 2, 3, 4, 5, and 6 mm of thickness of each attenuation square (or stack of squares) comprised of either native ABS or 2.7 g/cm^3^ ABS-bismuth composite. Region of interest analysis of each image was conducted using ImageJ software, and mean intensity values recorded in Microsoft Excel. Ratios were calculated at each thickness using ABS-bismuth values as the numerator and native ABS the denominator. Normalized ratios were then averaged with SEM (*n* = 4), plotted using GraphPad Prism and fitted to a single exponential decay to output half-life values as a measure of width of ABS-bismuth composite needed to block 50% of radiation from 99^m^Tc.

Radiation attenuation experiments for comparison of the 2.7 g/cm^3^ ABS-bismuth printed tube rack (shielded) relative to a standard tube rack (traditional) were conducted using ^99m^Tc solutions in standard 1.5 mL microfuge tubes. Sources were diluted to 224, 445, and 950 µCi activities in 500 µL of solution and measured immediately prior to experimentation using the same dose calibrator. Single tubes were placed in the front row of both the shielded and traditional tube racks and the transmitted gamma radiation was measured with a Geiger counter (Ludlum Measurements Inc., Sweetwater, TX, USA) placed 12 in from the edge of the tube rack. Tubes were then moved to the second row of their respective racks and measured again.

## 3. Results

### 3.1. Three Dimensional (3D) Printing Filaments to Mimic Human Tissues

The radiodensities of 11 different 3D-printed filaments were measured by taking CT scans of the filaments and quantifying the images using the Hounsfield Unit (HU) scale (Table 1). This scale is benchmarked with air at −1000 HU and water at 0 HU. Soft tissue has a radiopacity range of −100–100 HU [25,26] while bone has values of HU that typically measure 1000–3000 HU [27]. The chosen filaments ranged from polymers, such as ABS (natural and glow), ABS derivatives (Bendlay, IGUS iglidur), and PLA (polylactic acid, natural and glow), to polymer composites, such as Laybrick and woodFill, to metal-polymer composites, such as copperFill, bronzeFill, and GMASS bismuth (GMASS).

The filaments had a wide range of radiodensities, with the polymers having lower values and the metallic composite filaments having higher ones. Based on the results, ABS was chosen to represent soft tissue since it had a radiodensity in the middle of the expected range. However, none of the measured filaments matched the expected radiodensity of bone of 1000–3000 HU.

**Figure 1:**
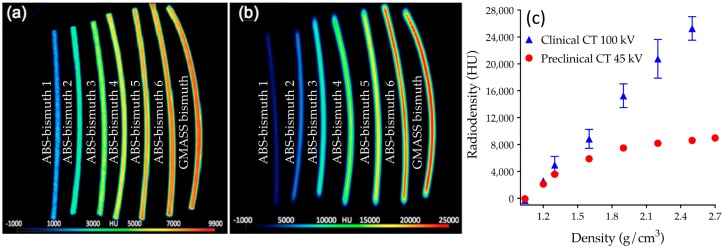
Radiodensities of 3D-printed filaments: Reconstructed image slice of preclinical CT (**a**) and clinical CT (**b**) data of ABS-bismuth and GMASS filaments with colorimetric representation of radiodensity; (**c**) Volume of interest data of radiodensities were plotted as a function of filament density for ABS, ABS-bismuth, and GMASS filaments from both preclinical and clinical CT scans.

In order to fabricate a filament that more closely emulated bone, a range of bismuth-infused ABS filaments was created by diluting GMASS in increasing amounts of ABS. The radiodensity of these filaments was measured on both a preclinical CT and a clinical CT system, along with standard ABS and GMASS filaments (Table 2, Figure 1).

**Figure 2:**
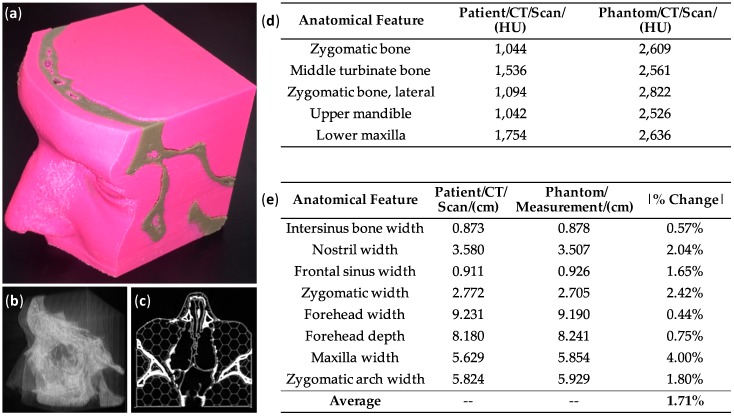
CT imaging and model accuracy of 3D-printed nasocranial phantom: (**a**) 3D-printed, multi-material nasocranial phantom with ABS soft tissue (pink) and ABS-bismuth 1 bone (gray); (**b**) coronal slice of clinical CT scan of nasocranial phantom, with bone regions appearing bright white due to high radiopacity; (**c**) 3D digital rendering of CT scan of phantom, showing high contrast of bone regions; (**d**) radiodensity data from original patient CT scan and from 3D-printed phantom CT scan at five coordinated bone locations; (**e**) distance measurements taken from the original patient CT scan data and from the physical 3D-printed phantom at eight coordinated locations, along with the absolute value of the percent change in distance between the two measures.

As expected, the radiodensity increased with the increasing concentration of bismuth. ABS-bismuth 1, with a density of 1.20 g/cm^3^, was the only filament with a radiopacity in the range of 1000–3000 HU and was therefore the best candidate for bone in phantom models. A multi-material nasocranial phantom was 3D printed, where soft tissue was printed in ABS (pink color) and bone was printed in ABS-bismuth 1 (Figure 2a). A clinical CT scan of the nasocranial phantom showed the bone portion appearing at a higher contrast (whiter color on grayscale image), demonstrating the higher radiopacity of the ABS-bismuth 1 portion (coronal slice shown in Figure 2b). The CT scan image was then processed into a 3D digital rendering, clearly showing the bone portion of the nasocranial phantom (light) and its stark contrast with the soft tissue section (gray) (Figure 2c). The radiopacity of the bone tissue in the phantom was compared to the original patient CT scan data at five distinct, corresponding regions. All measurements on both datasets fell within the expected 1000–3000 HU range for bone (Figure 2d). The dimensional accuracy of the phantom was determined by measuring the printed model and comparing it to measurements from the original CT scan data. Overall, the average percent difference in dimensions between the model and the CT data was 1.71%, with a maximum difference of 4.00% (Figure 2e).

### 3.2. Three Dimensional (3D) Printing Filaments for Radiation Attenuation

**Figure 3:**
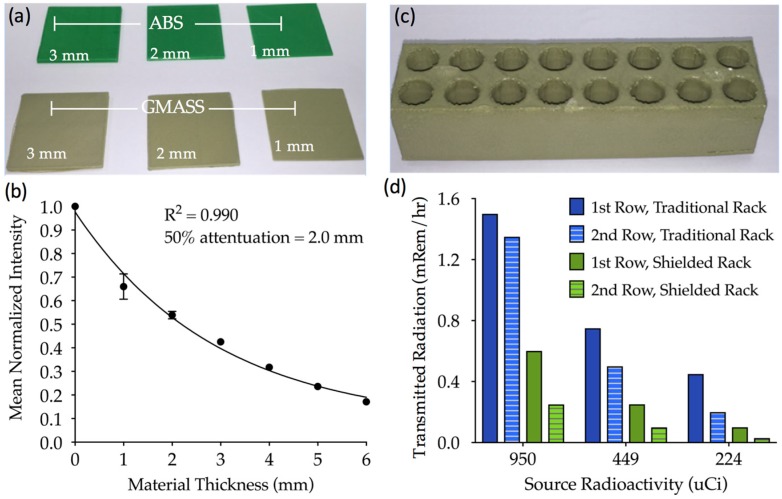
3D-printed GMASS attenuation of radiation from ^99m^Tc emission sources: (**a**) 3D-printed sheets made from ABS and GMASS filaments with thickness ranging from 1 to 3 mm that were stacked to make sheets with thickness between 1 and 6 mm; (**b**) Plot of mean normalized radiation intensity transmitted through GMASS sheets of varying thickness, normalized to ABS sheets of the same thickness. Each sheet thickness was subject to multiple radiation sources with distinct intensities, and an average normalized radiation intensity was calculated (black line); (**c**) 3D-printed test tube rack made from GMASS filament; Plot of transmitted radiation through the shielded GMASS tube rack and a traditional tube rack from ^99m^Tc emission sources with varying initial radioactivity levels.

While the radiopacity of filaments was titrated down by incorporating ABS, radiodense materials can attenuate high levels of radioactivity. Therefore, the GMASS filament was selected to quantify its ability to shield radiation. This was tested using different levels of a radioactive ^99m^Technetium (^99m^Tc) gamma emission source isotope and 3D-printed, GMASS squares of 1, 2, and 3 mm thickness (Figure 3a). The mean normalized radiation intensity decreased in an exponential fashion with the increasing GMASS sheet thickness, independent of the starting activity (Figure 3b). The four curves were averaged and fit to a one-phase exponential decay (R^2^ = 0.99), yielding a half-life value of 2.0 mm, indicating that this thickness of GMASS plastic will attenuate 50% of the ^99m^Tc radiation. A test tube rack was 3D printed from the GMASS filament, with a minimum wall thickness of 2 mm along the outer edge, to demonstrate the ability to print a customized, three-dimensional object capable of shielding a minimum of 50% of radiation from the ^99m^Tc isotope (Figure 3c). The ability of the shielded tube rack to attenuate radiation was compared to a traditional tube rack by placing ^99m^Tc radiation sources in multiple positions in each rack and measuring the transmitted radiation. The shielded rack transmitted between 60% and 85% less gamma radiation than the traditional rack, depending on the initial source radioactivity and the tube placement in either the first or second row of the respective racks (Figure 3d). The shielded rack attenuated a greater percentage of radiation as the source intensity decreased. Additionally, placing the source in the second row, thereby increasing the amount of GMASS between the source and the detector, reduced the transmitted radiation by an additional 60%–70% relative to source placement in the first row.

## 4. Discussion

With the wide range of materials currently available for 3D printing, many choices are available when constructing a phantom for CT imaging. However, in order to create a truly anthropomorphic device, it is necessary to select materials which mimic the imaging properties, such as X-ray opacity, of native tissues. However, there is not a thorough repository of radiation property data for 3D printing materials [23]. Furthermore, since various imaging techniques utilize different imaging energy levels, the materials must have the proper radiopacity at multiple incident energy levels. For instance, clinical CT systems use a higher energy imaging source than preclinical CT systems due to the need to penetrate larger human anatomy. Indeed, the clinical CT system showed a highly linear relationship between radiopacity and filament density using a 100 kVp source, as this energy level was able to penetrate the filaments with higher bismuth concentrations. The lower-energy 45 kVp preclinical system showed a linear relationship up to 1.3 g/cm^3^ of bismuth, followed by a plateau as the radiation source became almost completely attenuated. The ABS-bismuth 1 filament yielded HU values of 2113.0 and 2603.4 on the preclinical and clinical systems, respectively, making it the only filament tested that had imaging properties in the expected 1000–3000 HU range to emulate bone on both instruments. When creating a mimetic phantom it is important to note that native bone will not have a single value of radiopacity as it is heterogeneous tissue with varying densities.

The ABS-bismuth 1 filament was used in the fabrication of a 3D-printed, multi-material nasocranial phantom. The CT images and three-dimensional rendering of the CT scan both clearly showed the bone structure of the phantom, while the radiodensity measurements showed the strong correlation between the phantom and the original patient CT data, demonstrating that ABS-bismuth 1 is an appropriate imaging mimic for bone tissue. The dimensions of the printed model matched the original patient scan data with an average accuracy greater than 98%. This level of accuracy was expected because the resolution of the 3D printer is better than that of the original CT scan dataset. Because the ABS-bismuth 1 filament can be incorporated on consumer-grade 3D printers in facile fashion, it provides a promising option for creating accurate, patient-specific models. For example, the anthropomorphic nasocranial phantom could be used as a teaching aid for a CT technician to visualize the complex nasal structure due to its fidelity relative to the original patient data. Leary et al. noted the ability of high-accuracy phantoms to serve as better teaching models, thereby improving treatment outcomes [23]. Additionally, the tunability of the radiopacity makes it possible to use these ABS-bismuth filaments for 3D printing implantable materials, such as pins, plates, or screws, which would be straightforward to visualize during CT imaging. Additional research on other material properties, such as modulus, would be necessary to better understand the potential applications of ABS-bismuth 1.

In cases where the goal is to maximize radiation attenuation, as opposed to matching a radiopacity of a known tissue, increasing the bismuth concentration and overall density of the filament was found to be effective. As expected, the undiluted GMASS bismuth (2.7 g/cm^3^) filament had the highest radiopacity, meaning it was the optimal filament tested to shield against radiation transmission. Radiation shielding is important in labware, personal protective equipment, and equipment design to safeguard personnel from excessive radiation exposure. Therefore, we compared the ability of GMASS sheets to shield radiation from a common isotope used in biomedical single photon emission computed tomography (SPECT) imaging: ^99m^Tc with a gamma emission energy of 140 keV. Attenuation measurements were made and normalized to standard ABS to quantify the thickness of GMASS needed to block 50% of the ^99m^Tc radiation. The data in Figure 3 suggest that 2.0 mm of GMASS filament will attenuate half of the emission from this isotope relative to 2.0 mm of standard ABS. This makes it a strong candidate for radiation shielding of isotopes in this energy range or lower, especially for applications where easily printable and thin materials are required. A test tube rack that can be used to hold radioactive samples was 3D printed as a demonstration, but additional applications such as thin shielding for protective clothing or radiation source containment in imaging systems are also possible. It could also have clinical applications as 3D printing has been used to create customized radiation shields for a patient undergoing radiotherapy to protect surrounding healthy tissue [28,29].

Future work may entail further customization of the base GMASS filament to incorporate luminescent dyes or light-scattering nanoparticles to allow 3D-printed structures to be visible on a wider array of imaging devices, such as during optical fluorescence or luminescence acquisitions. Nanoprobes for multimodal biological imaging, including fluorescence, CT, and MR, have been synthesized and could serve as a starting point for multifunctional imaging dopants [30]. Thermoluminescent nanoparticles have been developed for use as radiotherapy dosimeters [31]. A GMASS filament doped with these particles could be used to train personnel on the CT equipment while simultaneously measuring the radiotherapy dose delivered to the phantom. The ability to customize the imaging properties of filaments shows promise for the future creation of myriad devices, objects, and implants that can be mass produced and finely tailored to suit specific needs within the medical space in a cost-effective manner.

## 5. Conclusions

The applications of 3D printing have been rapidly expanding as the underlying technology has matured, costs have decreased, and accessibility has increased. Three-dimensional printing for medical applications, particularly for medical imaging phantoms for training or calibration, has become an area of significant interest. To date, little research has focused on selecting printing materials that closely mimic the imaging properties of the native tissues they are being selected to represent. In this work, the initial focus was on characterizing one particular imaging property, the radiopacity, of different common 3D printing filaments as potential mimics for bone and soft tissue in a CT scan. ABS, a commonly utilized polymer, was found to be an appropriate soft tissue replica. In order to mimic bone, a commercially available bismuth-infused filament was diluted with ABS, demonstrating the ability to tune filament radiodensity and fabricate a bone mimetic material under CT. By combining ABS and bismuth-infused ABS, a nasocranial phantom was produced with the desired CT imaging properties. Additionally, maximizing the bismuth infusion created a filament that attenuated radiation to build objects such as protective labware or apparel. The ability to tune the radiopacity of a mainstream, printable filament opens up many future applications for uses within the medical imaging and lab safety spaces.

## Figures and Tables

**Table 1:** Radiodensities of 3D-printed filaments with standard deviations (SD). Filaments were imaged with a preclinical CT scanner and the data subsequently analyzed for HU values.

Filament	Radiodensity (HU)	SD (±)
Bendlay	−210.49	51.01
IGUS iglidur	−181.25	25.27
Natural ABS	−62.40	17.60
woodFill	14.73	31.40
Natural PLA	66.34	14.58
Glow ABS	156.15	46.04
Laybrick	256.30	9.43
Glow PLA	418.83	75.27
copperFill	8241.45	667.11
bronzeFill	8690.79	112.20
GMASS bismuth	8984.00	230.00

**Table 2:** Radiodensities of 3D-printed filaments. Filaments were imaged by both preclinical and clinical CT scanners and the images subsequently analyzed to obtain radiodensity values in HU.

Filament (Density, g/cm^3^)	Preclinical CT Radiodensity (±SD) (HU)	Clinical CT Radiodensity (±SD) (HU)
ABS (1.04)	−62.4 (17.6)	−282 (29.0)
ABS-bismuth 1 (1.20)	2113 (360)	2603 (374)
ABS-bismuth 2 (1.30)	3576 (280)	4960 (1278)
ABS-bismuth 3 (1.60)	5887 (221)	8848 (1408)
ABS-bismuth 4 (1.90)	7492 (264)	15,263 (1760)
ABS-bismuth 5 (2.20)	8193 (151)	20,759 (3883)
ABS-bismuth 6 (2.50)	8606 (250)	25,275 (1756)
GMASS (2.70)	8984 (230)	28,160 (1624)

## References

[1] Acharya R., Wasserman R., Stevens J., Hinojosa C. (1995). Biomedical imaging modalities: A tutorial. Comput. Med. Imag. Graph..

[2] Kalender W.A. (2006). X-ray computed tomography. Phys. Med. Biol..

[3] Yang K., Kwan A.L.C., Miller D.F., Boone J.M. (2006). A geometric calibration method for cone beam CT systems. Med. Phys..

[4] De Grand A.M., Lomnes S.J., Lee D.S., Pietrzykowski M., Ohnishi S., Morgan T.G., Gogbashian A., Laurence R.G., Frangioni J.V. (2006). Tissue-like phantoms for near-infrared fluorescence imaging system assessment and the training of surgeons. J. Biomed. Opt..

[5] Cotteleer M.J., Joyce J. (2014). D Opportunity: Additive Manufacturing Paths to Performance, Innovation, and Growth. Deloitte Rev..

[6] Gross B.C., Erkal J.L., Lockwood S.Y., Chen C., Spence D.M. (2014). Evaluation of 3D printing and its potential impact on biotechnology and the chemical sciences. Anal. Chem..

[7] Rengier F., Mehndiratta A., von Tengg-Kobligk H., Zechmann C.M., Unterhinninghofen R., Kauczor H.-U., Giesel F.L. (2010). 3D printing based on imaging data: Review of medical applications. Int. J. CARS.

[8] Bose S., Vahabzadeh S., Bandyopadhyay A. (2013). Bone tissue engineering using 3D printing. Mater. Today.

[9] Billiet T., Vandenhaute M., Schelfhout J., van Vlierberghe S., Dubruel P. (2012). A review of trends and limitations in hydrogel-rapid prototyping for tissue engineering. Biomaterials.

[10] Murphy S.V., Atala A. (2014). 3D bioprinting of tissues and organs. Nat. Biotechnol..

[11] Preece P., Williams S.B., Lam R., Weller R. (2013). “Let’s get physical”: Advantages of a physical model over 3D computer models and textbooks in learning imaging anatomy. Anat. Sci. Educ..

[12] Waran V., Narayanan V., Karuppiah R. (2014). Utility of multimaterial 3D printers in creating models with pathological entities to enhance the training experience of neurosurgeons. J. Neurosurg..

[13] Salmi M. (2016). Possibilities of preoperative medical models made by 3D printing or additive manufacturing. J. Med. Eng..

[14] Tuomi J., Paloheimo K.-S., Vehviläinen J., Björkstrand R., Salmi M., Huotilainen E., Kontio R., Rouse S., Gibson I., Mäkitie A.A. (2014). A novel classification and online platform for planning and documentation of medical applications of additive manufacturing. Surg. Innov..

[15] Salmi M. (2013). Medical Applications of Additive Manufacturing in Surgery and Dental Care. Ph.D. Thesis.

[16] Gear J.I., Long C., Rushforth D., Chittenden S.J., Cummings C., Flux G.D. (2014). Development of patient-specific molecular imaging phantoms using a 3D printer. Med. Phys..

[17] Burfeindt M.J., Colgan T.J., Mays O.R., Shea J.D., Behdad N., Van Veen B.D., Hagness S.C. (2012). MRI-derived 3-D-printed breast phantom for microwave breast imaging validation. IEEE Antennas Wirel. Propag. Lett..

[18] Wang J., Coburn J., Liang C.-P., Woolsey N., Ramella-Roman J.C., Chen Y., Pfefer T.J. (2014). Three-dimensional printing of tissue phantoms for biophotonic imaging. Opt. Lett..

[19] Carton A.-K., Bakic P., Ullberg C., Derand H., Maidment A.D.A. (2011). Development of a physical 3D anthropomorphic breast phantom. Med. Phys..

[20] Miller M.A., Hutchins G.D. Development of anatomically realistic PET and PET/CT phantoms with rapid prototyping technology. Proceedings of the IEEE Nuclear Science Symposium Conference Record.

[21] Cloonan A.J., Shahmirzadi D., Li R.X., Doyle B.J., Konofagou E.E., McGloughlin T.M. (2014). 3D-printed tissue-mimicking phantoms for medical imaging and computational validation applications. 3D Print Addit. Manuf..

[22] Wang M., Shen S., Yang J., Dong E., Xu R. (2014). 3D printing method for freeform fabrication of optical phantoms simulating heterogeneous biological tissue. Proc. SPIE.

[23] Leary M., Kron T., Keller C., Franich R., Lonski P., Subic A., Brandt M. (2015). Additive manufacture of custom radiation dosimetry phantoms: An automated method compatible with commercial polymer 3D printers. Mater. Des..

[24] 3D Slicer. http://pubs.acs.org.

[25] Thali M., Dirnhofer R., Vock P. (2009). The Virtopsy Approach: 3D Optical and Radiological Scanning and Reconstruction in Forensic Medicine.

[26] Molteni R. (2013). Prospects and challenges of rendering tissue density in Hounsfield units for cone beam computed tomography. Oral Surg. Oral Med. Oral Pathol. Oral Radiol..

[27] Schreiber J.J., Anderson P.A., Rosas H.G., Buchholz A.L., Au A.G. (2011). Hounsfield units for assessing bone mineral density and strength: A tool for osteoporosis management. J. Bone Jt. Surg. Am..

[28] De Beer D.J., Truscott M., Booysen G.J., Barnard L.J., van der Walt J.G. (2005). Rapid manufacturing of patient-specific shielding masks, using RP in parallel with metal spraying. Rapid Prototyp. J..

[29] Zemnick C., Woodhouse S.A., Gewanter R.M., Raphael M., Piro J.D. (2007). Rapid prototyping technique for creating a radiation shield. J. Prosthet. Dent..

[30] Zeng S., Tsang M.-K., Chan C.-F., Wong K.-L., Hao J. (2012). PEG modified BaGdF_5_:Yb/Er nanoprobes for multi-modal upconversion fluorescent, in vivo X-ray computed tomography and biomagnetic imaging. Biomaterials.

[31] Salah N., Khan Z.H., Habib S.S. (2011). Nanoparticles of Al_2_O_3_:Cr as a sensitive thermoluminescent material for high exposures of gamma rays irradiations. Nucl Instr. Methods Phys. Res. B.

